# A Case of Decreased Amplitude in Motor Evoked Potentials Under Remimazolam Anesthesia

**DOI:** 10.7759/cureus.27593

**Published:** 2022-08-02

**Authors:** Yuichi Aratani, Yasuyuki Tokinaga, Tadashi Tanioku, Tomoyuki Maruyama, Tomoyuki Kawamata

**Affiliations:** 1 Anesthesiology, Wakayama Medical University, Wakayama, JPN

**Keywords:** decreased amplitude, motor evoked potential, spine surgery, total intravenous anesthesia, remimazolam

## Abstract

Remimazolam is a newly developed benzodiazepine derivative. Although one case report on the use of remimazolam for motor evoked potential (MEP) monitoring has been reported, there has been no report of changes in the MEP response under remimazolam anesthesia, which is associated with impairment of the corticospinal motor track. This is a case of a 54-year-old woman who was diagnosed with an extradural extramedullary tumor. The patient reported being allergic to chicken eggs. We used remimazolam instead of propofol for anesthesia management. During tumor resection, the amplitudes of MEP responses at the left quadriceps femoris, left tibialis anterior, and left abductor hallucis muscle decreased. The surgery was scaled down and the tumor was removed in a reduced size. The patient had muscle weakness immediately after surgery but eventually recovered. In this case, we could detect changes in MEP response under remimazolam anesthesia, which suggested impairment of the motor tracts during surgery.

## Introduction

Remimazolam is a newly developed benzodiazepine derivate that has favorable pharmacological properties for anesthetic management including rapid onset of action and reversal of its pharmacological effects by flumazenil [1]. Remimazolam (launched by Mundipharma) was approved in 2020 in Japan for use in general anesthesia. Time to loss of consciousness was 102 seconds and 88.7 seconds for 6 mg/kg/hour and 12 mg/kg/hour dosages, respectively [2].

Motor evoked potentials (MEPs) are often used to assess spinal motor function during spinal surgery. If the amplitude of the MEP is reduced by 75%, the sensitivity and specificity of predicting postoperative neurologic deficit are 94% and 94%, respectively [3]. Because most anesthetics other than opioids suppress the MEP response [4], anesthesiologists must pay attention to the type and dose of anesthetics. Benzodiazepines including midazolam can be used during MEP monitoring [5]. However, there has been only one report [6] on the use of remimazolam for MEP monitoring. Here, we report the intraoperative MEP responses in a patient who underwent spine surgery under general anesthesia using remimazolam. We could detect changes in MEP response under remimazolam anesthesia, which suggested impairment of the motor tract during surgery.

## Case presentation

A 54-year-old woman was diagnosed with an extradural extramedullary tumor on close examination of lumbar and buttock pain. She was 163.8 cm tall and weighed 70.7 kg with a body mass index of 26.4 kg/m^2^. She was allergic to chicken eggs, and there was no other medical history (American Society of Anesthesiologists physical status 2). Because preoperative magnetic resonance imaging (MRI) revealed a suspected tumor at the Th11 to Th12 level, Th11 and 12 laminectomy and tumor resection were scheduled. Before surgery, she did not have motor paralysis but had numbness in her left thigh. Because she had a history of egg allergy, we decided to use remimazolam instead of propofol for anesthesia management.

General anesthesia was induced with remimazolam at 12 mg/kg/hour and remifentanil at 0.2 μg/kg/minute. Following confirmation of loss of consciousness, remimazolam was administered at 1 mg/kg/hour. Tracheal intubation was performed after muscle relaxation had been achieved with 40 mg of rocuronium. During surgery, remimazolam was administered at 0.9 mg/kg/hour, and the bispectral index was maintained at 40-60. Remifentanil was administered at 0.3 μg/kg/minute, and no additional muscle relaxants were administered.

**Figure 1:**
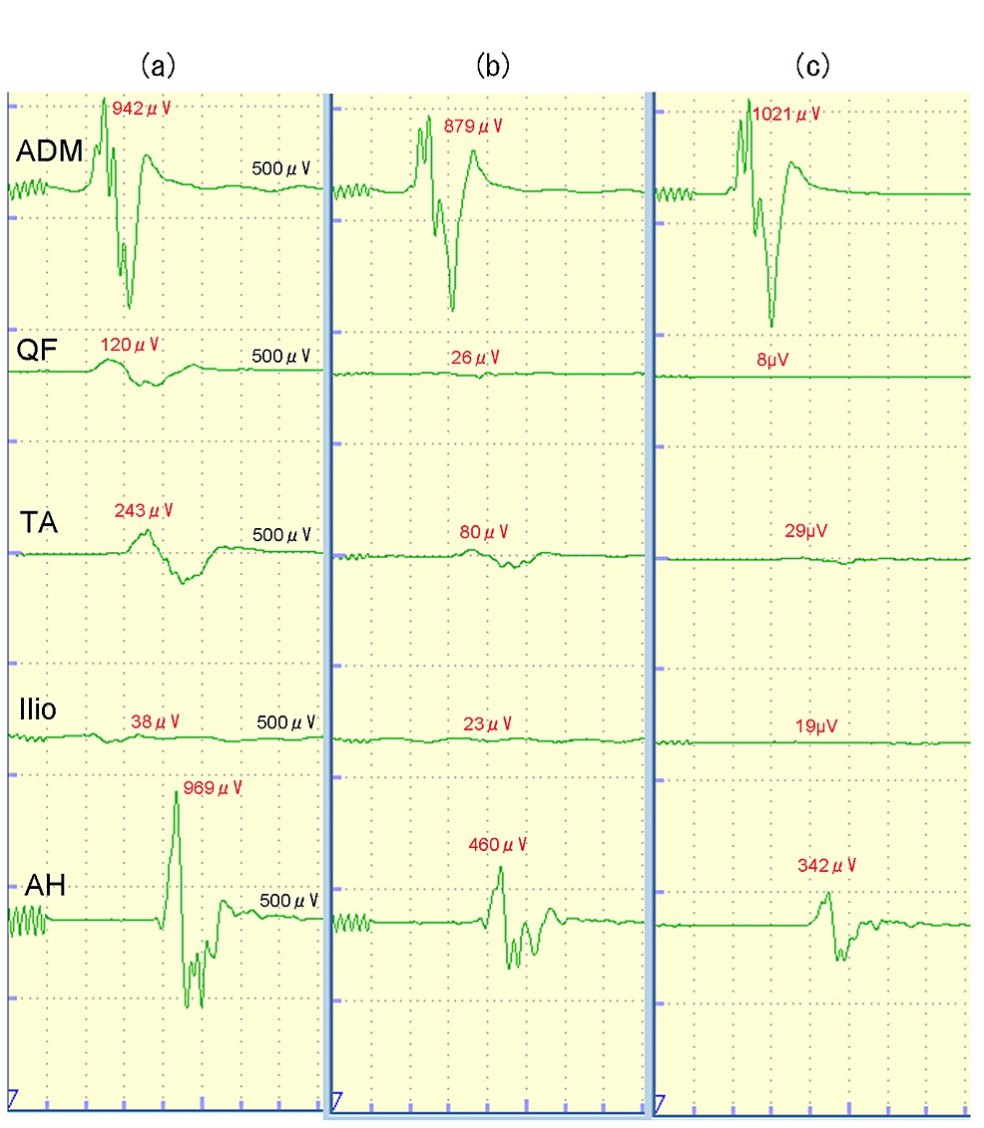
MEP responses from the left extremities. MEP responses from the left extremities (a) before surgery, (b) during, and (c) at the end. MEP: motor evoked potential; ADM: abductor digiti minimi; QF: quadriceps femoris; TA: tibialis anterior; Ilio: iliopsoas muscle; AH: abductor hallucis muscle

MEP responses were recorded at the abductor digiti minimi (ADM), quadriceps femoris (QF), tibialis anterior (TA), iliopsoas muscle (Ilio), and abductor hallucis muscle (AH). Transcranial electrical stimulation for MEPs was performed using train-of-five pulses with 2-ms intervals at 500 V and 200 mA (Neuromaster neurophysiological monitoring system, Nihon Kohden, Tokyo, Japan). The amplitude of the MEP response was determined by the peak-to-peak amplitude, and the latency was measured from the start of stimulation to the onset of myogenic activity. Recording of the MEP response was considered successful if the recorded amplitude was 50 μV or higher per trial. A 50% decrease in the amplitude of the MEP response was assessed as a significant change. Before surgery, the MEP response elicited by intracranial stimulation was successfully monitored at each muscle. MEP responses at each muscle during tumorectomy and at the end of surgery were comparable to those before surgery (Figure 1). After antagonizing the effect of rocuronium with sugammadex (200 mg), baseline MEP responses before surgery were recorded. MEP response elicited by intracranial stimulation was successfully monitored at each muscle. During tumor resection, the amplitudes of MEP responses at the left QF, left TA, and left AH decreased to 6.7%, 12.0%, and 38.4% of the basal values, respectively (Figure 1). The amplitude of the MEP response at the left ADM was comparable to the baseline value. Although the surgery was temporarily interrupted, the reduced amplitudes of MEP responses at the left QF, left TA, and left AH were not improved. The surgery was scaled down. The tumor was removed in a reduced size. The amplitudes of MEP responses remained decreased until the end of surgery. The operative time was 4 hours and 41 minutes.

After emergence from anesthesia, she was unable to kneel on the left side and complained of numbness in her left lower abdomen. One day after the surgery, her motor impairment and numbness in her left lower abdomen were improved. There was no change in the numbness in her left thigh that was present before the surgery. She was discharged from the hospital 19 days after the surgery with no further problems.

## Discussion

Although both intravenous anesthetics and volatile anesthetics suppress the amplitude of MEPs, intravenous anesthetics produce significantly less depression [7]. Propofol has been preferred for total intravenous anesthesia because of its short-acting nature compared to that of conventional benzodiazepines including midazolam and diazepam [8]. The newly developed benzodiazepine remimazolam has a shorter context-sensitive half-time (predicted to be 7 ± 2 minutes after a four-hour infusion) than that of propofol [9]. The amplitude of MEPs decreased in our patient, and weakness of the foot was confirmed after anesthesia because of the rapid elimination of remimazolam. Remimazolam has some advantages including no vascular pain at administration and less circulatory depression [10]. In addition, remimazolam can be used for patients with a history of egg allergy. However, there has been only one report on the effects of remimazolam on MEP response [10]. Our case report provides further evidence regarding the use of remimazolam for MEP monitoring. In our patient, we were able to detect MEP responses at a clinically used dose of remimazolam (0.9 mg/kg/hour). We were also able to detect a decrease in the amplitude of the MEP response with surgical manipulation, which was considered to be associated with motor impairment observed after recovery from general anesthesia. In our patient, we detected unilateral decreases of MEP responses in the left QF, TA, and TH during tumor resection. The MEPs of the QF, which plays an important role in knee standing, were greatly depressed. Our patient was unable to kneel on the left side in the early postoperative period. A decreased MEP response has been reported to be caused by the effects of anesthetics, decreased body temperature, anemia, and hypotension [11-13]. These causes are false positives for a decreased MEP response. An example of a false positive is anesthetic fade [14]. An anesthetic fade is commonly observed in all MEP waveforms being recorded. In our case, an anesthetic fade is unlikely to have been involved in the decreased MEP response. There was no hypothermia, anemia, hypotension, dislodged electrodes, or mechanical problems when MEP response depression occurred. When we consider the postoperative course, the decreased MEP response in our case was most likely due to the detected abnormalities in the motor tract.

Finally, because this is only a case report, further studies are needed to determine the indications for remimazolam in surgery using MEP monitoring.

## Conclusions

We could detect changes in MEP response under remimazolam anesthesia, which suggested impairment of the motor tract during surgery. Remimazolam may be an alternative anesthetic to propofol in surgeries where MEPs are measured if the patient has a contraindication to propofol.
